# Increased Levels of Cortisol in Individuals With Suicide Attempt and Its Relation With the Number of Suicide Attempts and Depression

**DOI:** 10.3389/fpsyt.2022.912021

**Published:** 2022-06-10

**Authors:** Alma Delia Genis-Mendoza, Diana María Dionisio-García, Thelma Beatriz Gonzalez-Castro, Carlos Alfonso Tovilla-Zaráte, Isela Esther Juárez-Rojop, María Lilia López-Narváez, Rosa Giannina Castillo-Avila, Humberto Nicolini

**Affiliations:** ^1^Laboratorio de Genómica de Enfermedades Psiquiátricas y Neurodegenerativas, Instituto Nacional de Medicina Genómica, Ciudad de México, Mexico; ^2^Hospital Psiquiátrico Infantil “Juan N. Navarro”, Servicios de Atención Psiquiátrica, Mexico City, Mexico; ^3^División Académica de Ciencias de la Salud, Universidad Juárez Autónoma de Tabasco, Villahermosa, Mexico; ^4^División Académica Multidisciplinaria de Jalpa de Mendéz, Universidad Juárez Autónoma de Tabasco, Jalpa de Méndez, Mexico; ^5^División Académica Multidisciplinaria de Comalcalco, Universidad Juárez Autónoma de Tabasco, Comalcalco, Mexico; ^6^Secretaria de Salud de Chiapas, Hospital Chiapas Nos une “Dr. Jesús Gilberto Gómez Maza”, Tuxtla Gutiérrez, Mexico

**Keywords:** suicide attempt, cortisol, HPA axis, suicide behavior, stress, suicide

## Abstract

**Background:**

Abnormalities in the hypothalamic-pituitary-adrenal axis (HPA) have been reported in individuals with suicide behavior. The aim of the study was to evaluate cortisol levels in peripheral plasma of individuals with more than one suicide attempt.

**Methods:**

Cortisol concentrations in peripheral plasma were measured using the ELISA technique. Suicide attempts were evaluated by the Columbia Suicide Severity Rating Scale, while depression was evaluated by the Hamilton Depression Rating Scale.

**Results:**

We found elevated cortisol levels in the suicide attempt group when compared with healthy controls (*F* = 7.26, *p*-value = 0.008), but no statistical differences with the psychiatric diseases group (*F* = 1.49, *p*-value = 0.22). Cortisol levels were higher in individuals with depression (*F* = 8.99, *P* = 0.004) and in individuals with two or more suicide attempts (*F* = 13.56, *P* < 0.001).

**Conclusions:**

Cortisol levels are increased in individuals who attempt suicide and higher of cortisol concentrations in plasma regard to depression and more attempts of suicide.

## Introduction

Suicide is a public health problem worldwide. In 2020, 1.5 million deaths by suicide were reported (1). Epidemiology studies indicate that an estimate of 80% of these deaths were in low and middle-income countries (2, 3).

The etiology of suicide behavior is unknown; however, it has been proposed a diathesis-stress model to explain its pathogenesis. It is believed that the diathesis may have a biological origin. The literature shows that, when stress in perceived, there are clear changes in cortisol levels (4). Therefore, the stress diathesis theory of suicide postulates that there are predisposing and precipitating risk factors of suicide attempts. Genetic, inflammation, serotoninergic systems, peripheral biomarkers as cholesterol and, the alteration of the hypothalamic-pituitary-adrenal axis (HPA), could be participant in the biological diathesis of suicide behavior (5–7).

Some studies have addressed the association between altered levels of circulating cortisol and an increased risk of suicide behaviors (8); nonetheless, these studies have observed a variety of outcomes. For example, in a response to an experimental stressor in suicide attempters compared with non-suicidal individuals, a low baseline cortisol prior to the stressor was found in attempters (9). However, in veterans with and without suicide attempts, a no-significant association with cortisol levels and suicidal behavior was observed (10); similarly, no-significant association with cortisol levels was observed in individuals with psychiatric illnesses and suicide behavior (11, 12) also, higher cortisol levels were associated with increased suicidal severity and suicide attempts (13) moreover, increased cortisol response after dexamethasone suppression test have been presented in patients that were weary life (14) and in Due to the inconsistent results regarding the association of cortisol levels and suicide behavior, two meta-analyses have explored this association (15, 16) and their results suggest a possible role of cortisol levels in suicide behavior. Therefore, the need of more studies performed in other populations and the inclusion of healthy and psychiatric controls is imperative in order to determine the role of cortisol levels and suicide attempt. The objectives of the present study were to evaluate plasma cortisol levels in Mexican individuals with suicide attempt and to determine if cortisol levels differ between these individuals and those with psychiatric diseases and a healthy comparison group; and finally, to associate plasma cortisol levels with the characteristics of suicide attempt.

## Methods

This is a cross-sectional study conducted from January to December, 2020, in Tabasco, Mexico. This study meets the criteria established in the Declaration of Helsinki and was approved by the ethics committee of the Regional Hospital of High Specialty of Mental Health (HRAESM/DG/UWI/351/2020). All participants signed an informed consent. None of the individual included in the study received financial compensation for their participation.

### Participants

Our study group comprised a total of 143 Mexican individuals, that were recruited from February 2020 to July 2021. We included three hospitals in Tabasco State: The High Specialization Regional Hospital Dr. Gustavo A. Rovirosa Pérez in Villahermosa City, The General Hospital of Comalcalco Dr. Desiderio G. Rosado Carbajal in Comalcalco City, and The Regional Hospital of High Specialty of Mental Health in Villahermosa City.

The sample was divided in 3 groups: (1) The suicide attempt group included 56 patients who had a diagnosis of suicide attempt, made by at least one psychiatrist using the evaluation of suicide behavior by the Columbia Suicide Severity Rating Scale (C-SSRS); they were all selected from admissions to the hospital emergency department. (2) The group of individuals with psychiatric diseases (excluding depression) and no-history of suicide behavior was formed by 31 patients; the psychiatric diagnoses (including schizophrenia) were made by at least one psychiatrist. (3) The healthy control group included 56 participants who reported no-history of suicide attempt or psychiatric illnesses. All the participants were volunteers; they were informed about the study, the procedure and the objectives; they all signed an informed consent form and agreed to the collection of the biological sample. The objective of including these three groups was to compare the cortisol levels in three types of population, since literature indicates variations among them.

### Data Collection

Demographic characteristics such as age, sex, occupation, marital status, educational level, sample collection time, BMI (body mass index) and substance use were gathered. Additionally, we included information about their suicide behavior using the C-SSRS; to evaluate symptoms of depression we used the Hamilton Depression Scale (HAM-D). In the suicide attempt group, we also enquired about the family history of suicide attempts, number of suicide attempts, method of suicide attempt, as well as age of first and last suicide attempts.

### Individuals With Suicide Attempt

The clinical diagnosis was made by at least one psychiatrist according to the DSM-5 (Diagnostic and Statistical Manual of Mental Disorders 5) criteria for determining suicide attempt, and they used the C-SSRS (which is validated in Spanish and increases the ability to predict suicidal risk) full version including 16 questions with binary responses. The C-SSRS is used as an initial screening to guide clinicians in assessing suicide risk and to help stratify patients into categories of low, moderate or high lifetime patient risk. The C-SSRS is formed by four sub-scales: severity and intensity of suicidal ideation and severity and lethality of suicide behavior (17); and 20 categories: wish to be dead, with no-specific active suicidal thoughts; active suicidal ideation, with any methods, without intent to act; active suicidal ideation, with some intent to act, without specific plans; active suicidal ideation, with specific plan and intent; preparatory suicide acts or suicide behavior; aborted suicide attempt; interrupted suicide attempt; actual suicide attempt (non-fatal); and completed suicide. If one question is answered “yes”, in categories 1–5 is suicidal ideation, in categories 6–10 is suicide behavior. In the same way, information about the number of suicide attempts was collected, age when the first suicide attempt occurred, age of last suicide attempt, family history of suicide attempts and whether they suffer from any psychiatric illnesses.

### Psychiatric Individuals Without Suicide Attempt

The group of individuals with psychiatric diseases but without suicide attempt were diagnosed by psychiatrics according to the DSM-5. Who reported in the interviews that they did not have any previous suicide attempts and we corroborated that information in their medical records.

### Control Group

The control group was formed by 56 individuals (64.3% males and 35.7% females) with an age range of 18–55 years and a mean of 34.30 years of age (±10.37), who were volunteer donors from the blood bank of The High Specialization Regional Hospital Dr. Gustavo A. Rovirosa Pérez. This group received a general check-up by physicians and they reported no psychiatric illness or history of suicide attempts.

### Assessment of Depression

Depression symptoms were evaluated in individuals with suicide attempt using the HAM-D, consisting of 17 questions validated in Spanish and evaluates the depressive symptomatology of the patients (18). It was important to evaluate depression, since it is an important factor related to suicide attempts (19). The scale is widely available and has two common versions with either 17 or 21 items that score between 0 and 4 points. The first 17 items measure the severity of depressive symptoms and as examples the interviewer rates the level of agitation clinically noted during the interview or how the mood is impacting on an individual's work or leisure pursuits. The extra four items on the extended 21-point scale measure factors that might be related to depression, but are not thought to be measurements of severity, such as paranoia or obsessional and compulsive symptoms. The scoring is based on the 17-item scale and scores of 0–7 are considered as being normal, 8–16 suggest mild depression, 17–23 moderate depression and scores over 24 are indicative of severe depression; the maximum possible score is 52 on the 17-point scale.

### Cortisol Samples

Peripheral blood samples were taken from three groups and the blood was placed in EDTA (Ethylenediamine tetraacetic acid) containing tubes [BD Vacutainer, K2 EDTA (K2E)] Plus Blood Collection Tubes, placed on ice. Samples from the group of individuals with suicide attempt were taken the day after their admission to the emergency room. After the peripheral blood samples were collected, they were immediately centrifuged and plasma aliquots were made and stored at −80°C. Cortisol concentrations in plasma were determined by means of the competitive Enzyme-Linked Immunoabsorption Assay (ELISA). The samples were analyzed using a commercially available Cortisol Competitive Human ELISA kit by Invitrogen. The mean of sample collection was at 11:00 am.

### Data Analyses

Data were analyzed using the SPSS version 27.0 (IBM Corporation, Armonk, NY). Descriptive variables were expressed as means and Standard Deviation (SD) for continues variables, and frequencies and percentages for categorical variables. The initial comparison between patients with suicide attempt and the comparison groups were analyzed with *X*^2^-test also for categorical variables and with independent samples *t*-test for continuous variables. In continues variables, an analysis of variance followed by Bonferroni correction were performed. Cortisol levels and confounding variables were analyzed by linear regression analysis. We performed linear regression with confounding variables (age, sex, education, marital status, socioeconomic level, time of collection and body mass index) which did not present statistical differences that influenced the cortisol levels of our study group. To determine the association between cortisol levels and suicide attempt, we performed an age-adjusted linear regression analysis. Significance was established at *p* ≤ 0.05 for all analyses.

## Results

### Sociodemographic Characteristics

In the Table 1 are shown the characteristics of the study population. The majority in the overall population were men *n* = 79 (55.6%) and unemployed *n* = 75; the majority were not-married *n* = 74 (51.7%) and had studied more than 6 years *n* = 116 (81.1%). As for substances use, most of them did not consume alcohol *n* = 75 (52.4%), neither cannabis *n* = 133 (93%) nor smoke cigarettes *n* = 123 (88.35%).

**Table 1:** Sociodemographic characteristics of suicide attempters, patients with psychiatric diseases and healthy individuals in a Mexican population.

**Characteristics**	**All** ***n* = 143**	**Healthy controls** ***n* = 56**	**Psychiatric patients** ***n* = 31**	**Suicide attempt** ***n* = 56**	**Statistics**
**Sex**					
Males	80 (55.6)	36 (64.3)	19 (61.3)	25 (43.6)	*X*^2^ = 5.30, ***p*** **=** **0.07**
Females	63 (44.4)	20 (35.7)	12 (38.7)	31 (56.4)	
**Occupation**					
Unemployed	23 (16.1)	3 (5.4)	9 (29.0)	11 (19.6)	*X*^2^ = 42.62, ***p*** **<** **0.001**
Home	36 (25.2)	8 (14.3)	15 (48.4)	13 (23.2)	
Student	16 (11.2)	5 (8.9)	1 (3.2)	10 (17.9)	
Part-time employment	25 (17.5)	9 (16.1)	4 (12.9)	12 (21.4)	
Full-time employment	43 (30.1)	31 (55.4)	2 (6.5)	10 (17.9)	
**Marital status**s					
Married	61 (42.7)	41 (73.2)	3 (9.7)	17 (30.4)	
Single	74 (51.7)	12 (16.2)	27 (87.1)	35 (62.5)	
Widowed	1 (0.7)	1 (1.8)	0	0	*X*^2^ = 43.34, ***p*** **<** **0.001**
Separated/divorced	7 (4.9)	2 (3.6)	1 (3.2)	4 (7.1)	
**Education**					
<6 year of schooling	27 (18.9)	10 (17.9)	10 (32.3)	7 (12.5)	*X*^2^ = 5.14, *p* = 0.76
>6 years of schooling	116 (81.1)	46 (82.1)	21 (67.7)	49 (87.5)	
**Alcohol consumption**					
Yes	68 (47.6)	35 (62.5)	3 (9.7)	30 (44.1)	*X*^2^ = 23.66, ***p*** **<** **0.001**
No	75 (52.4)	21 (37.5)	28 (37.3)	26 (34.7)	
**Cannabis use**					
Yes	10 (7)	0	2 (6.5)	8 (14.3)	*X*^2^ = 8.8, ***p*** **=** **0.01**
No	133 (93)	56 (100.0)	29 (93.5)	48 (85.7)	
**Cigarette smoking**					
Yes	16 (11.2)	0	5 (16.1)	11 (19.6)	*X*^2^ = 11.84, ***p*** **=** **0.003**
No	127 (88.8)	56 (100)	26 (83.9)	45 (80.4)	

*Numbers in bold show significant statistical difference*.

The suicide attempt group included more women *n* = 31 (56.4), unemployed *n* = 34 (60.7%), mostly single *n* = 39 (69.6%), and with an education of more than 6 years *n* = 49 (87.5). The majority consumed alcohol *n* = 30 (44.1), but not cannabis *n* = 45 (85.7%) and no cigarette smoking *n* = 44 (80%), and the mean cortisol level was 14.68 μg/dL (SD 15.36).

The psychiatric diseases group included more men *n* = 19 (61.3%), single *n* = 27 (87.1%), and who had more than 6 years of schooling *n* = 21 (67.7%). Most of them, did not consume alcohol *n* = 28 (90.3%), neither cannabis *n* = 29 (93.5%) and no cigarette smoking *n* = 24 (82.8%).

In the healthy control group there were more men *n* = 36 (64.3%), unemployed *n* = 16 (64%), and married *n* = 41 (73.2%). The majority had studied more than 6 years *n* = 46 (82.1%). As for the substances abuse, most of them used alcohol *n* = 35 (62.5%) but not cannabis *n* = 56 (100%) and no cigarette smoking *n* = 56 (100%).

Comparison of groups using the cortisol levels are show in the Table 2. We observed difference between groups. In accordance with *post-hoc* comparisons, healthy controls and suicide attempt groups reported statistically significant. Since, the suicide attempt groups showed a higher cortisol levels that healthy controls groups. However, the *post-hoc* analysis of healthy controls and psychiatric controls not differences were observed.

**Table 2:** Comparison of cortisol levels and age among groups.

	**All** ***n* = 143**	**Healthy controls** ***n* = 56**	**Psychiatric patients** ***n* = 31**	**Suicide attempt** ***n* = 56**	**Statistics**		***Post-hoc*** **comparison *p*-value (mean difference)**		
		**Mean (SD)**	**Mean (SD)**	**Mean (SD)**	***F***	***p*** **-value**	**HC vs. SA**	**HC vs. PP**	**PP vs. SA**
Cortisol level	11.97 ± 11.29	8.79 ± 6.74	12.79 ± 7.36	14.68 ± 15.36	**4.08**	**0.01**	**0.01 (5.89)**	0.32 (4.00)	1 (1.89)
Age	32.88 ± 11.04	34.30 ± 10.37	40.25 ± 9.50	32.50 ± 11.65	5.35	**0.006**	1 (−1.80)	0.13 (−5.95)	**0.05 (7.75)**

*HC, Healthy controls; PP, Psychiatric patients; SA, Suicide attempts*.

*Numbers in bold show significant statistical difference*.

### Characteristics of the Individuals With Suicide Attempt

Table 3 shows the characteristics of the suicide attempts in the cases group, most of them had one suicide attempt *n* = 37 (66.1%), did not have familiar history of suicide attempts *n* = 24 (75%), the mean age of the first suicide attempt was at 29.28 years old ±12.90 and the last suicide attempt was at 31.64 years old ±13.82. Where the characteristics were compared between men and women, there were no statistical differences, in our study group sex was not related to suicide phenotypes.

**Table 3:** Characteristics of suicide attempt among males and females in Mexican population.

	**All**	**Males**	**Females**	**Statistics**
**Number of SA**				
1	37 (66.1)	14 (58.3)	23 (71.9)	*X*^2^ = 1.12, *p* = 0.29
2 or +	19 (33.9)	10 (41.7)	9 (28.1)	
**Familiar history of SA**				
Yes	8 (25.0)	4 (33.3)	4 (20.0)	*X*^2^ = 0.71, *p* = 0.39
No	24 (75.0)	8 (66.7)	16 (80.0)	
**Age of the first SA**	29.28 ± 12.90	31.04 ± 14.33	27.96 ± 11.77	*F* = 0.43, *p* = 0.39
**Age of the last SA**	31.64 ± 13.82	33.08 ± 14.08	30.56 ± 13.74	*F* = 0.17, *p* = 0.50
**Depression**				
Yes	14 (25)	5 (20)	9 (29.0)	*X*^2^ = 0.60, *p* = 0.43
No	42 (75)	20 (80)	22 (70.9)	

*SA, suicide attempts*.

### Levels of Plasma Cortisol (μg/dL) in Subgroups of Suicide Attempters and Comparison Groups

In Table 4, we show the levels of cortisol in plasma μg/dL) in subgroups of suicide attempters and the comparison groups (healthy individuals or with psychiatric diseases) in a Mexican population.

**Table 4:** Level of plasma cortisol (μg/dL) in subgroups of suicide attempters and comparison groups (health and psychiatric group) in a Mexican population.

**Groups**	***n***	**Age Mean ±SD**	**Cortisol Mean ±SD**	**Statistics** ***F***	***P-*** **value**
**Controls**	56	32.55 ± 11.75	8.79 ± 6.74	Reference	
**Psychiatric controls**	31	43.71 ± 13.40	12.79 ± 7.36	5.53	**0.02**
**Suicide attempted**	56	34.30 ± 10.37	14.68 ± 15.36	7.26	**0.008**
**PC vs. SA**	56	43.71 ± 13.40	14.68 ± 15.36	1.49	0.22
**SA-depression**					
No	42	33.14 ± 12.15	8.79 ± 6.74	5.17	**0.02**
Yes	14	30.78 ± 10.67	18.32 ± 20.54	8.99	**0.004**
**SA-number of attempt**					
One	37	31.21 ± 12.44	13.42 ± 16.50	4.10	**0.04**
2 or +	19	35.15 ± 10.09	17.15 ± 12.76	13.56	**<0.001**

*PC, psychiatric controls; SA, suicide attempted; SD, standard deviation*.

*Depression by Hamilton Scale Depression. Statistically significant are present in bold*.

The results of the linear regression analysis were statistically significant when we compared cortisol levels between healthy controls and individuals with suicide attempt (*F* = 7.26, *P*-value = 0.008) (Table 3). But no statistically significant differences were observed when comparing with the psychiatric diseases group (*F* = 1.49, *p* = 0.22). Subsequently, we evaluated differences in the presence of symptoms of depression. We found that individuals with (*F* = 8.99, *P* = 0.004) and without depression showed increased cortisol levels in comparison with healthy controls *and/or* individuals with psychiatric diseases.

## Discussion

In this study, we evaluated plasma cortisol levels in individuals with suicide attempt in comparison with two groups, healthy individuals and individuals with psychiatric diseases (excluding depression). Up to date, research has not delivered conclusive outcomes that determine if cortisol levels are decreased or increased in individuals who attempt suicide (15, 20, 21). We also analyzed cortisol levels according to clinical and sociodemographic variables; to our knowledge, this is the first study that evaluates cortisol levels in Mexican individuals who have attempted suicide.

We found significantly elevated cortisol levels in the suicide attempt group in comparison with the healthy control group. Our results agree with other studies that have observed high cortisol levels in suicide attempt groups when compared with individuals without suicide behavior (22–29). Sociodemographic characteristics of cases and comparison groups in our population studied were very similar; however, their circulating cortisol levels differed significantly. Hence, we could assume that cortisol levels are related with an alteration of the stress regulation system in patients with suicide attempt (30). In the case of the individuals with psychiatric diseases (excluding depression), cortisol levels were not significantly different when compared with the suicide attempt group. Nevertheless, both groups had higher cortisol levels then the healthy individuals group. Some studies have concluded that individuals with psychiatric diseases (excluding suicide behavior and depression) show different cortisol levels than patients with suicide behavior (15). Other reports found that subjects with psychiatric disorders have shown HPA axis hyperactivity and alterations in cortisol levels (31, 32); furthermore, individuals with suicide attempt could have a more active HPA axis than individuals with other psychiatric disorders (11, 12).

On the other hand, we observed statistical differences when we analyzed the characteristics of the suicide attempt group associated with cortisol levels such as the presence or absence of depression. Significantly elevated cortisol levels were observed in the group of individuals who had attempted suicide, in comparison with the baseline cortisol levels of the healthy group, as some previous studies have recorded (23, 27, 33). Our results suggest that cortisol levels could be a phenotype of suicide attempters because of an alteration in the HPA axis, and this could be increased in the presence of depression or the number of suicide attempts.

A conclusive description of the increased levels of cortisol in patient with suicide attempt has not been reached yet. However, some explanations have been proposed: some studies correlate 5-HT1A receptors of serotonin (5-HT) with cortisol levels, stress and suicide attempt (34, 35). In this sense, the altered serotonin is associated with suicide behavior, making people more aggressive and more susceptible to mood disorders (36, 37). Also, the hippocampus plays an important role in feedback mechanisms of the HPA axis (38, 39), as well as in the levels of 5HT1A receptors. The hyperactivity of the HPA axis produced by chronic exposure to stress generates an elevation of plasma corticosteroids, including cortisol (40). Having high levels of cortisol reduces the mRNA of 5HT1A receptors, decreasing their levels, resulting in low rates of serotonin metabolism, but when serotonin dysfunction occurs there is a risk of suicidal behavior (41–43). Another way in which high cortisol levels have been related to a decrease in 5-HT, is due to the increased tryptophan 2,3-dioxugenase (TDO) (20, 21); this enzyme is involved in the degradation of tryptophan (TRP) *via* the kynurenine pathway, which synthesizes 5-HT (44). With lower levels of TRP, there is not enough TRP available in brain to synthesize 5-HT, as there are more enzymes that metabolize TRP (45), and decreased amounts of the 5-HT neurotransmitter are associated with attempted suicide (46, 47).

The HPA hyperactivity in our group with psychiatric diseases is presumed to be different from our suicide attempt group, because one of the factors that contribute in the development of suicide ideation and suicide attempt is the presence of psychiatric illness (48–50) such as schizophrenia disorder (51, 52). In our results, suicide attempt group and psychiatric control group showed high cortisol levels, which indicates that there was hyperactivity of the HPA axis in both groups. But even if there were no significant differences between these groups, the suicide attempt group showed the highest cortisol levels. We assumed that this is a duality, where apart from suicidal behavior, a psychiatric illness is present, and both situations increased cortisol levels in patients who attempted suicide. Considering that cortisol levels in suicide attempt subjects were much higher when in presence of psychiatric illness and suicide ideation, it was important for us to include a control group with psychiatric illness to see any difference in cortisol levels. Our results support that we can use cortisol as a particular biomarker of suicidal attempt.

Some limitations in this study should be noted. First, we did not determine menstrual cycle, contraceptive use or hormone replacement in women who participated in the study. Second, the sample size could be considered small. However, it is the first study that explores cortisol levels in a Mexican population with suicide attempt. Third, this is a cross sectional study, then longitudinal studies that measure cortisol levels are needed. Also, we did not include the use of medications and if they received psychotherapy or some medication. Further, we did not aboard if the study population had been received psychological treatment in their childhood, neither, the evaluation of resilience capacity, or if they had some stressful life events, among other evaluations that could have been of great value for our study to include.

In conclusion, we found increased levels of plasma cortisol in individuals with suicide attempt. The level of cortisol is higher in presence of depression and in individuals with more than two attempts suicide. Then, levels of cortisol in individuals who attempt to die by suicide could be considered when searching for a biomarker of suicide attempts and become part of the preventing measures of this global problem.

## Data Availability Statement

The original contributions presented in the study are included in the article/Supplementary Material, further inquiries can be directed to the corresponding authors.

## Ethics Statement

The studies involving human participants were reviewed and approved by HRAESM/DG/UWI/351/2020. Written informed consent to participate in this study was provided by the participants' legal guardian/next of kin.

## Author Contributions

Conceptualization, writing-original draft preparation, and supervision: DD-G, TG-C, CT-Z, AG-M, IJ-R, ML-N, and RC-A. Methodology: DD-G, TG-C, CT-Z, and IJ-R. Formal analysis and investigation: DD-G, TG-C, and CT-Z. Editing: DD-G, TG-C, CT-Z, and RC-A. All authors contributed to the article and approved the submitted version.

## Conflict of Interest

The authors declare that the research was conducted in the absence of any commercial or financial relationships that could be construed as a potential conflict of interest.

## Publisher's Note

All claims expressed in this article are solely those of the authors and do not necessarily represent those of their affiliated organizations, or those of the publisher, the editors and the reviewers. Any product that may be evaluated in this article, or claim that may be made by its manufacturer, is not guaranteed or endorsed by the publisher.
