# Supplementary material for: Increased Levels of Cortisol in Individuals With Suicide Attempt and Its Relation With the Number of Suicide Attempts and Depression
Source: Front Psychiatry. 2022 Jun 10;13:912021. doi: 10.3389/fpsyt.2022.912021 (PMC9226316; doi:10.3389/fpsyt.2022.912021)
Supplement: Supplementary file 1 [file Table_1.DOCX]

Supplementary Material

**Supplementary Table 1.** Cortisol levels and confounding variables.

|  | ***R*** | **P value** | **F** | **P value** |
| --- | --- | --- | --- | --- |
| **Age** | 0.09 | 0.27 | 1.25 | 0.17 |
| **Sex** | 0.07 | 0.39 | 0.71 | 0.39 |
| **Education** | 0.11 | 0.16 | 0.74 | 0.77 |
| **Marital status** | 0.09 | 0.27 | 1.45 | 0.23 |
| **Socioeconomic level** | -0.013 | 0.87 | 0.64 | 0.52 |
| **Sample collection time** | 0.039 | 0.64 | 1.06 | 0.39 |
| **BMI** | -0.07 | 0.37 | 0.34 | 1 |
